# Water Evaporation and Conformational Changes from Partially Solvated Ubiquitin

**DOI:** 10.1155/2010/213936

**Published:** 2010-10-11

**Authors:** Saravana Prakash Thirumuruganandham, Herbert M. Urbassek

**Affiliations:** Fachbereich Physik und Forschungszentrum OPTIMAS, Technische Universität Kaiserslautern, Erwin-Schrödinger-Straße, 67663 Kaiserslautern, Germany

## Abstract

Using molecular dynamics simulation, we study the evaporation of water molecules off partially solvated ubiquitin. The evaporation and cooling rates are determined for a molecule at the initial temperature of 300 K. The cooling rate is found to be around 3 K/ns, and decreases with water temperature in the course of the evaporation. The conformation changes are monitored by studying a variety of intermediate partially solvated ubiquitin structures. We find that ubiquitin shrinks with decreasing hydration shell and exposes more of its hydrophilic surface area to the surrounding.

## 1. Introduction

Common mass-spectrometric techniques for biomolecules take their starting point from analyte molecules embedded in a matrix—such as MALDI (matrix-assisted laser desorption and ionization)—or solvated in a liquid; here examples are the ESI (electrospray ionization) or LILBID (laser-induced liquid beam ion desorption) techniques. Ideally, of course, mass spectrometry is applied only to the bare biomolecule, after it has been stripped of all its solvent molecules. The last stages of this liberation process are of particular relevance as they will determine the effective speed of this process and the final conformation of the biomolecule. A recent review article [1] outlines that the desolvation process of proteins embedded in *μ*m-size droplets encompasses several stages and the induced conformation changes of the protein evolve along with the dehydration process. Here, the evaporation of water from the droplet, which lets it shrink and cools it, until the protein is fully dehydrated, is only the first step; it is followed by the *“hydrophobic collapse”* of the charged side chains of the dry protein, occurring in a time scale of a few ps, as a consequence of the loss of the hydration shell. On longer time scales of milliseconds, gas-phase collisions with surrounding molecules as well as radiative reheating may have further effects on the protein conformation, such as a loss of hydrophobic bonds and dissociation of electrostatic interactions. The folding into more stable gas-phase structures may finally proceed until the seconds- or minutes-time scale. Molecular-dynamics (MD) simulation can be employed to elucidate the first stages of these processes, that is, the dehydration and evaporative cooling steps, as well as the associated conformation changes; these early processes are not easily accessible to experiment.

A number of computational studies using MD simulations of partly solvated proteins in vacuum [2–7] or of desolvation dynamics [8, 9] have recently been reported. Among the MD studies devoted to the unfolding and refolding behaviour of gas phase and solvated proteins, we mention [10–12], which are devoted to cytochrome c and lysozyme. The protein ubiquitin was investigated in [2, 3, 13–15] with a focus on conformation changes in solution and thermal denaturization conditions.

 In this paper, we shall analyze the final stages of evaporation of water from a ubiquitin protein. We consider a rather cold protein, which starts at 300 K and evaporatively cools down to around 270 K. We monitor the conformation changes of the molecule by considering a number of intermediate, partially solvated structures.

## 2. Method

We consider a ubiquitin molecule (a small globular protein found in all eukaryotes consisting of 76 amino acid residues, a total of 13222 atoms with a molecular weight of 8433 Da) in 5 different environments:

1. bulk water realized as a cubic water volume with periodic boundary conditions,
2. a water droplet of 806 water molecules (called *thick shell)*,
3. a water droplet of 279 water molecules (called *thin shell)*,
4. vacuum (no water),
5. crystalline ubiquitin, with the pdb structure [16].

**Figure 1:**
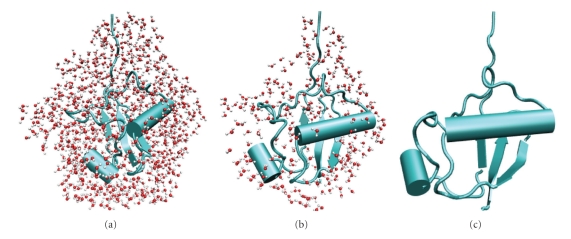
Perspective view of equilibrated ubiquitin-water complexes for evaporation simulation at 300 K. (a) Thick hydration shell. (b) Thin hydration shell. (c) Vacuum structure.

The structures 2, 3 and 4 are visualized in Figure 1. In this study, only the neutral charge state is considered; it corresponds to a pH 7 water environment [3]. We note that in a low pH environment, ubiquitin becomes highly charged (+13 charges at pH 2); it then unfolds to the so-called A state [15].

The bulk water system is realized by setting the biomolecule in a cubic water volume of 50 Å side length with periodic boundary conditions. Before starting the evaporation simulation, we prepare the system as follows. We remove so-called bad water-protein contacts—that is, water molecules whose oxygen atom is closer than 2.3 Å to protein nonhydrogen atoms were removed. The system is then subjected to energy minimization using the steepest descent (SD) and adopted basis set Newton-Raphson (ABNR) modules of CHARMM to relax the molecular system. Later, a short 50 ps MD simulation is used with harmonic constraints on the protein to allow solvent relaxation. After the minimization, the system is subjected for 50 ps to a slow progressive heating using the Berendsen algorithm to the working temperature of 300 K. Finally, the system is equilibrated during 150 ps in a constant volume and temperature MD simulation. This system contains 3997 water molecules.

**Figure 2:**
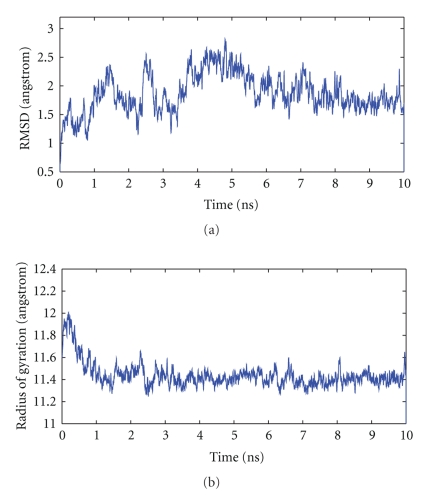
Structural drift of the protein ubiquitin in bulk water at 300 K during a 10 ns constant temperature and pressure simulation. Result of a single simulation run. (a) Root mean square deviation of C_*α*_ backbone. (b) Radius of gyration.

This bulk-water system is simulated for 10 ns in a constant-temperature (300 K) and constant-pressure (1 bar) ensemble. The mass of the pressure piston and Langevin piston collision frequency were set to 400 amu and 20 ps^−1^, respectively.  Figure 2 demonstrates that at least in the last 3-4 ns, the protein has assumed an equilibrium conformation, while in the first several ns, the larger fluctuations in the RMSD indicate conformational changes.

We use this equilibrated structure to prepare samples 2–4 as follows. The thin- (thick-) shell structure is prepared by cutting away all water molecules with a distance of more than 3 (6) Å from the protein. The number of water molecules amounts to 279 (806) for these systems. After preparing these droplets, we couple them to a 300 K heat bath for 10 ps, and then let them relax in free dynamics for another 40 ps to find their equilibrium structures.

The last structure, no. 5, (crystalline ubiquitin) is taken from the pdb, including its native water (58 molecules). Only a single protein with its native water molecules is simulated at 300 K in a vacuum environment; three replicas are generated, which correspond to thermal fluctuations of this system. This structure has been included for reference purposes.

For modelling the dynamics, we have used version c31b of the CHARMM MD software tool [17]. We note that different parameterizations of the force fields may in principle yield different simulation results; however, a previous study—on the structural stability of various electrosprayed proteins, among them ubiquitin, against thermal and hydration changes—found only little influence of the force field employed (OPLS-AA/L, AMBER03, GROMOS96) [14]. Water is described using the TIP3P potential [18] in the CHARMM version [19]. Investigations using different rigid-water models for studies of water clusters in vacuum have shown that the choice of the water model does only little influence evaporation rates and cooling [20]; this conclusion was corroborated in a recent study of water evaporation from ubiquitin-containing droplets [14]. We employ a Verlet integrator scheme to calculate the dynamics with a time step of Δ*t* = 1 fs. The SHAKE algorithm [21] is used for all hydrogen atoms.

The results given below are averages over 3 statistically uncorrelated starting structures; their water content varies by ±7 molecules from the average values given above. This admittedly small number of statistically independent runs is not untypical of evaporation studies [14] and appeared sufficient for our conclusions to be drawn. These structures are put into vacuum for a 10 ns simulation of water evaporation. After this time, temperatures have been reduced to below 275 K. While it is known that evaporation proceeds until a temperature of 215 K [20] on a *μ*s time scale, the evaporation rates become exceedingly small with decreasing temperature.

## 3. Results

### 3.1. Temperature and Evaporation

**Figure 3:**
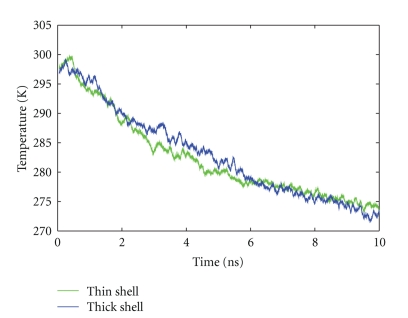
Temperature of ubiquitin during the evaporation process. Data are averaged over 3 simulation runs; the statistical error is ±2 K, Table 1.

Figure 3 demonstrates the evaporative cooling of the protein over a period of time of 10 ns. For both the thin-shell and the thick-shell system we observe a cooling rate of around 3 K/ns. A closer look shows that the initial cooling is faster, around 4.3 ± 0.4 K/ps, while at later times, when the temperature has dropped below 280 K, the cooling rate amounts to only 1.3 K/ps. The thin-shell system appears to be cooled slightly faster at the beginning; this would be in line with the smaller heat capacity of this system, but this conclusion is at the limit of the reliability of the data. In terms of evaporated molecules, the thin-shell system lost around 15% of its water shell, while the thick-shell system lost 9%, Table 1.

A previous simulation [3] studied the evaporation of water droplets enclosing ubiquitin in a scenario analogous to ours, but in a different force field (OPLS-AA), and a TIP4P water model. Their results indicate a temperature drop of 33 (36) K for the analogue of our thick-shell (thin-shell) system; a fraction of 9 (15)% of the water molecules were evaporated. The satisfying agreement shows that the evaporation and cooling process is robust with respect to the simulation model employed. We note that our data for the thin-shell system are also in satisfactory agreement with the results of [14], who obtained (for the equivalent of our thin-shell system) 10% loss at 275 K, and 29% loss at 325 K, both for a 15 ns evaporation period.

The cooling rate is roughly identical for the thick- and the thin-shell system, giving evidence that the system temperature, rather than a changed conformation, is the main parameter controlling evaporation. The evaporation rate amounts to 4 (7) molecules/ns for the thin- (thick-) shell droplet, Table 1. These data can be compared to an extensive study of evaporation from pure water clusters in vacuum [20], performed for water clusters containing between 125 and 4096 molecules with starting temperatures between 250 and 300 K. That investigation monitored evaporation up to times of 3 *μ*s; they showed that clusters evaporate until their temperature has decreased to around 220 K, demonstrating that evaporation continues after our simulation time of 10 ns. This study found that the relative change in temperature of a cluster is linearly correlated to the relative change in the number of molecules. For our starting temperature of 300 K that study predicts a relative fraction of evaporated molecules of 4.2%; this value is definitely smaller than the values of 15% and 9% found in this study, Table 1. This difference must be attributed to the different geometry (and content) of the water clusters: while our clusters contain a ubiquitin protein and all water sits at the surface, the clusters of [20] contain pure water, thus reducing the *relative* number of evaporated molecules.

### 3.2. Protein Conformation

We measured the ubiquitin conformation using the root mean square deviation (RMSD) of the C_*α*_ backbone structure (with the fully solvated 300 K system as reference), the radius of gyration *R*_gyr_ and the number of hydrogen bonds within the protein, HB_pp_, and between protein and water, HB_ps_. For identifying the hydrogen bonds, we use a distance criterion (*r* < 3.5 Å) between hydrogen and acceptor and accept a maximum angle of 30° for the hydrogen-donor-acceptor angle. In addition, the solvent accessible surface area (SASA) and the solvent excluding surface area (SESA) [22–25] were determined. These areas are meant to provide information about the hydrophilic (SASA) and hydrophobic (SESA) parts of the protein. To measure SASA, a probe sphere is rolled over the van-der-Waals surface of the protein; SASA is the area traced out by the centre of the probe sphere during this motion. The radius of the probe sphere is taken as 1.4 Å, corresponding to the radius of a water molecule. SESA is the area of the corresponding surface which is not accessible to the probe sphere, due to steric hindrance by other parts of the protein. In this work, we have calculated the SASA of the protein using the module developed by Lee and Richards [22] and implemented in CHARMM [26]. SESA was calculated as the difference between the SASA and the van-der-Waals surface area, cf, for example, [3].

Rather than monitoring these quantities in the evaporating droplet, the measurements were performed on the 5 systems introduced above, and have been averaged over 3 ns simulation of an equilibrium system; this provides better statistical accuracy. The results are given in Table 2. They show a clear trend: by reducing the hydration shell the protein conformation continuously evolves from the fully hydrated structure to the vacuum structure. The steady decrease of the radius of gyration indicates a compactification (shrinkage) of the fully hydrated protein to its dry conformation. As the increase of the SESA—and the concomitant decrease of the SASA—indicates, the molecule exposes a larger part of its previously hidden hydrophobic surface towards the vacuum; due to the loss of the hydration water this is not penalized energetically. The number of hydrogen bonds towards the solvent decreases with decreasing solvation shell; this is plausible since the number of bonding partners decreases. The number of hydrogen bonds within the protein is rather stable, demonstrating that the intraprotein hydrogen network is largely conserved while ubiquitin loses its hydration shell.

We note that a recent study of ubiquitin conformation changes under electrospray conditions [3] obtained results analogous to ours. Their simulations were performed using the OPLS-AA force field and a TIP4P water model. The good agreement proves that our conclusions are robust with respect to the simulation model employed.

**Figure 4:**
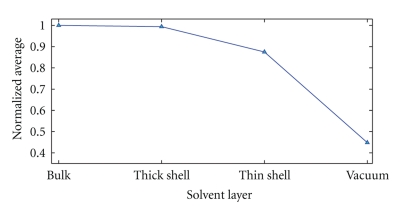
Normalized average of hydrogen bonds of ubiquitin in various aqueous environments. The sum of protein-protein and protein-water bonds, normalized to the value for the bulk system, is shown. The statistical error is ±0.02, Table 2.

Figure 4 displays how the total number of hydrogen bonds (normalized to the value for a fully hydrated protein in the bulk-water system) evolves with decreasing hydration. The sum of protein-protein and protein-water bonds is shown. Evidently, the thick hydration shell is undistinguishable from the fully hydrated protein; this can be taken as evidence that this thick shell is sufficient to give the protein its bulk-water conformation. The thin hydration shell, however, shows around 15% deviations from the fully hydrated value. The fully dehydrated vacuum protein shows the largest difference, Table 2. This is in agreement with the general finding that during the evaporation process, even a small amount of remaining water is able to stabilize the solution structure, while major conformational changes are about to happen only for the fully isolated protein [1].

## 4. Conclusions

In summary, we determined an evaporation rate of around 4–7 water molecules/ns from partially solvated ubiquitin. During the simulated evaporation time of 10 ns, it cools down from 300 K to around 270–275 K. Evaporative cooling proceeds at a rate of around 3 K/ns. It is faster at 300 K (4.3 K/ns) and slows down with decreasing temperature such that it is only 1.3 K/ns at 280 K. With decreasing hydration shell, the conformation of ubiquitin changes continuously from the fully solvated structure to the vacuum structure: the protein shrinks slightly and exposes more of its hydrophilic surface area to the vacuum. The hydrogen bonding network shows definite changes with respect to the bulk structure: the largest changes with respect to the bulk-water structure are observed for the (fully dehydrated) vacuum structure; the changes disappear when a second hydration shell of 6 Å width has been added to the molecule.

## Figures and Tables

**Table 1:** Absolute number *N*_*evap*_ and fraction *N*_*evap*_/*N* of water molecules that have evaporated after simulations of 10 ns. Average temperature *T* of the final protein-water cluster, averaged over the last 0.5 ns of the simulations.

System	*N*_evap_	*N*_evap_/*N* (%)	*T* (K)
Thin shell	43 ± 5	15.3 ± 1.5	274 ± 2
Thick shell	69 ± 3	8.6 ± 0.4	273 ± 2

**Table 2:** Comparison of structural properties of the protein in various environments, averaged over the last 3 ns of the simulation. Except for the bulk water simulation, averages over 3 simulations are shown. The crystal simulations show almost vanishing fluctuations, except for the RMSD.

	Bulk water	Thick shell	Thin shell	Vacuum	crystal
RMSD [Å]	1.8	2.4 ± 0.3	2.7 ± 0.3	2.4 ± 0.1	2.6 ± 0.4
SASA [nm^2^]	49.7	47.2 ± 0.4	45.9 ± 0.02	41.9 ± 0.2	42.8
SESA [nm^2^]	28.8	29.2 ± 0.1	30.5 ± 0.2	31.1 ± 0.2	31.6
*R*_gyr_ [Å]	11.93	11.57 ± 0.06	11.49 ± 0.03	11.36 ± 0.05	11.28
HB_pp_	229.3	230.7 ± 3.1	227.1 ± 3.2	216.9 ± 5.5	223.4
HB_ps_	254.8	250.5 ± 5.4	194.4 ± 3.7	—	87.5

## References

[1] Breuker K, McLafferty FW (2008). Stepwise evolution of protein native structure with electrospray into the gas phase, 10–12 to 102 s. *Proceedings of the National Academy of Sciences of the United States of America*.

[2] Alonso DOV, Daggett V (1998). Molecular dynamics simulations of hydrophobic collapse of ubiquitin. *Protein Science*.

[3] Patriksson A, Marklund E, van der Spoel D (2007). Protein structures under electrospray conditions. *Biochemistry*.

[4] Steinberg MZ, Breuker K, Elber R, Gerber RB (2007). The dynamics of water evaporation from partially solvated cytochrome c in the gas phase. *Physical Chemistry Chemical Physics*.

[5] Hamaneh MB, Buck M (2007). Acceptable protein and solvent behavior in primary hydration shell simulations of hen lysozyme. *Biophysical Journal*.

[6] Sykes MT, Levitt M (2007). Simulations of RNA base pairs in a nanodroplet reveal solvation-dependent stability. *Proceedings of the National Academy of Sciences of the United States of America*.

[7] Zhou L, Siegelbaum SA (2008). Effects of surface water on protein dynamics studied by a novel coarse-grained normal mode approach. *Biophysical Journal*.

[8] Pande VS, Rokhsar DS (1999). Molecular dynamics simulations of unfolding and refolding of a *β*-hairpin fragment of protein G. *Proceedings of the National Academy of Sciences of the United States of America*.

[9] Fernández A (2002). Desolvation shell of hydrogen bonds in folded proteins, protein complexes and folding pathways. *FEBS Letters*.

[10] Mao Y, Woenckhaus J, Kolafa J, Ratner MA, Jarrold MF (1999). Thermal unfolding of unsolvated cytochrome c: experiment and molecular dynamics simulations. *Journal of the American Chemical Society*.

[11] Woenckhaus J, Mao YI, Jarrold MF (1997). Hydration of gas phase proteins: folded +5 and unfolded +7 charge states of cytochrome c. *Journal of Physical Chemistry B*.

[12] Reimann CT, Velázquez I, Bittner M, Tapia O (1999). Proteins in vacuo: a molecular dynamics study of the unfolding behavior of highly charged disulfide-bond-intact lysozyme subjected to a temperature pulse. *Physical Review E*.

[13] Koeniger SL, Merenbloom SI, Sevugarajan S, Clemmer DE (2006). Transfer of structural elements from compact to extended states in unsolvated ubiquitin. *Journal of the American Chemical Society*.

[14] Marklund EG, Larsson DSD, Van Der Spoel D, Patriksson A, Caleman C (2009). Structural stability of electrosprayed proteins: temperature and hydration effects. *Physical Chemistry Chemical Physics*.

[15] Segev E, Wyttenbach T, Bowers MT, Gerber RB (2008). Conformational evolution of ubiquitin ions in electrospray mass spectrometry: molecular dynamics simulations at gradually increasing temperatures. *Physical Chemistry Chemical Physics*.

[16] Berman HM, Westbrook J, Feng Z (2000). The protein data bank. *Nucleic Acids Research*.

[17] Brooks BR, Brooks CL, Mackerell AD (2009). CHARMM: the biomolecular simulation program. *Journal of Computational Chemistry*.

[18] Jorgensen WL, Chandrasekhar J, Madura JD, Impey RW, Klein ML (1983). Comparison of simple potential functions for simulating liquid water. *The Journal of Chemical Physics*.

[19] MacKerell AD, Bashford D, Bellott M (1998). All-atom empirical potential for molecular modeling and dynamics studies of proteins. *Journal of Physical Chemistry B*.

[20] Caleman C, Van Der Spoel D (2006). Temperature and structural changes of water clusters in vacuum due to evaporation. *Journal of Chemical Physics*.

[21] Ryckaert J-P, Ciccotti G, Berendsen HJC (1977). Numerical integration of the cartesian equations of motion of a system with constraints: molecular dynamics of n-alkanes. *Journal of Computational Physics*.

[22] Lee B, Richards FM (1971). The interpretation of protein structures: estimation of static accessibility. *Journal of Molecular Biology*.

[23] Richards FM (1977). Areas, volumes, packing and protein structure. *Annual Review of Biophysics and Bioengineering*.

[24] Bizzarri AR, Cannistraro S (2002). Molecular dynamics of water at the protein-solvent interface. *Journal of Physical Chemistry B*.

[25] Pascual-Ahuir JL, Silla E, Tuñón I (1998). The solvent-excluding surface as a descriptor of ionic channels: Gramicidin-A. *Journal of Molecular Structure: THEOCHEM*.

[26] Jarrold MF (2000). Peptides and proteins in the vapor phase. *Annual Review of Physical Chemistry*.

